# Guide to recent advances in difference-in-differences methodology for population health studies

**DOI:** 10.1136/jech-2025-225449

**Published:** 2026-06-25

**Authors:** Matt Sutton, Igor Francetic, Stephen O’Neill, Samuel Hugh-Jones, Luke Munford

**Affiliations:** 1 Health Organisation, Policy and Economics, Centre for Primary Care and Health Services Research, School of Health Sciences, University of Manchester, Manchester, UK; 2 Department of Health Services Research and Policy, London School of Hygiene & Tropical Medicine, London, UK

**Keywords:** HEALTH POLICY, PUBLIC HEALTH, RESEARCH DESIGN

## Abstract

**Background:**

Difference-in-differences is a method commonly used in population health research. It is based on assumptions that are well-known and well-understood in the classic textbook example of two groups (exposed and unexposed) and two periods (before and after). However, several papers have emerged recently highlighting further complications, for instance, when there are multiple groups and/or multiple periods and/or variation in timing of exposure across groups. Many ways to adjust the classic difference-in-differences methodology have been proposed.

**Methods:**

We explain the challenges for difference-in-differences methodology in real-world settings. We summarise alternative methods that have been proposed to deal with deviations from the classic difference-in-differences situation and set out some considerations for choosing between estimators.

**Results:**

The straightforward reasoning of the difference-in-differences method cannot be assumed when the effects of an exposure vary between groups and/or vary over time. These so-called ‘heterogeneous treatment effects’ require explicit consideration because typical ways to estimate difference-in-differences make implicit assumptions about how to create a summary measure of impact from multiple comparisons. We explain how proposed alternative methods deal with the aggregation of these heterogeneous treatment effects.

**Conclusions:**

Real-world applications of the difference-in-differences method typically require the analyst to allow the effects of an exposure to vary across treatment groups and over time. Summary assessment of impact requires an explicit weighting of heterogeneous impacts. The preferred approach to difference-in-differences depends on how exposure is assigned and the availability of data on exposed and unexposed groups before and after exposure begins.

**WHAT IS ALREADY KNOWN ON THIS TOPIC**

- Difference-in-differences is a popular method in population health studies.
- The method is often explained using two groups (exposed and unexposed) and two periods (before and after).
- Real-world population health problems are rarely this simple.
- Application of new difference-in-differences methods is required for these problems.

**WHAT THIS STUDY ADDS**

- A non-technical explanation of when simple difference-in-differences methods may not be valid.
- Brief introductions to two popular new approaches using doubly robust or extended regression methods.
- Advice on applying difference-in-differences methods to real-world situations.

**HOW THIS STUDY MIGHT AFFECT RESEARCH, PRACTICE OR POLICY**

- Increased awareness of the challenges in real-world settings could improve difference-in-differences studies.
- Detailed exploration and stronger justification of fundamental assumptions are needed.
- Researchers should test the sensitivity of their results to alternative difference-in-differences approaches.

## Background

 Difference-in-differences has become a widely used approach in population health research. It is applicable when there is information on the outcome of interest for at least one exposed and one unexposed (or comparator) group both before and after the exposure began for the exposed group. The design is typically explained^1^ using a 2×2 table with the difference-in-differences estimate capturing how the change in outcome over time differs between the group that becomes exposed (or ‘treatment’ group) and the group that remains unexposed (or ‘control’ group) (see Box 1).

Box 1 **The classic 2×2 difference-in-differences example**

Classic difference-in-differences is often presented using a 2×2 table that compares two groups (exposed vs unexposed) across two time periods (before vs after).

Definition of terms:

- Group E is exposed and receives the treatment.
- Group U is unexposed and does not receive the treatment.
- The ‘pre’ or ‘before’ period is period 0. Neither group has been exposed.
- The ‘post’ or ‘after’ period is period 1. Only the exposed group has been exposed.

The average outcomes (Y) are:

- $Y_{E,0}$ for the exposed group in the before period.
- $Y_{E,1}$ for the exposed group in the after period.
- $Y_{U,0}$ for the unexposed group in the before period.
- $Y_{U,1}$ for the unexposed group in the after period.

We want to estimate the *causal effect* of the intervention on the exposed group, known as the average treatment effect on the treated (ATT).

A naïve comparison would be the change over time in the group that becomes exposed:

$Y_{E,1}-Y_{E,0}$

However, this ignores any changes over time that would have happened anyway.

One possible solution is to use the change over time in the unexposed group to adjust for these trends. If we are willing to assume that the change over time in the exposed group would have been the same as the change over time in the unexposed group if the exposed group had not been exposed, we can use a difference-in-differences estimator.

The difference-in-differences estimator

$\text{DiD}=(Y_{E,1}-Y_{E,0})-\left(Y_{U,1}-Y_{U,0}\right)$

where:

- $Y_{E,1}-Y_{E,0}$ (the first difference) is the change over time for the treatment group.
- $Y_{U,1}-Y_{U,0}$ (the second difference) is the change over time for the control group.
- Subtracting the second difference from the first difference removes common trends, isolating the treatment effect under the ‘parallel trends assumption’.

Interpretation

If the control group represents what would have happened to the treatment group in the absence of treatment (the ‘parallel trends assumption’), then difference-in-differences gives an unbiased estimate of the treatment effect.

The ability of the difference-in-differences method to produce an unbiased estimate of the effect of an intervention relies on key assumptions: no anticipation, parallel trends, sufficient overlap and no spillovers.^2^ There are established ways to examine the plausibility of these assumptions, some by conceptual reasoning and some by empirical testing.

However, the simple 2×2 setting rarely applies in practice, and real-world population health studies are more complex. For example, attempts to evaluate the mental health impacts of a major social welfare reform in the UK called Universal Credit have revealed several complications. Mental health is influenced by many factors other than social welfare reform that vary over time and space. The introduction of Universal Credit that most researchers have evaluated is the staggered process across geographical areas between 2014 and 2019.^3^ This welfare reform potentially impacts all people who might be entitled to this benefit or to the benefits that it replaced. Implementation issues are likely to lead to impacts that vary between cohorts starting to be exposed at different times. The initial impact of being placed on Universal Credit is likely different from the longer-term impacts.

Recent papers in the econometrics literature have clarified that the elegant simplicity of difference-in-differences only applies to the 2×2 case. If there is more than one exposed group and/or more than one period after exposure begins, then the effect of the exposure may vary, and this heterogeneity of impact is often a key concern for researchers. Moreover, recent papers have shown that ignoring this heterogeneity can lead to misleading results.

Several of the new approaches to difference-in-differences estimation that address the requirements of evaluation in the real world have been surveyed and compared in several recent reviews in econometric^4 5^ and in health journals.^6 7^ Our aim in this paper is to explain the limitations of simple difference-in-differences intuitively in applied population health settings and provide guidance on how to navigate the multitude of new estimators and approaches that are now available in the literature and in standard analysis software.

## Methods

We begin by repeating the fundamental characteristics and assumptions of the simple 2×2 design. We then describe deviations from this simple situation that are often encountered in practice and explain why these cause problems for the traditional way of estimating difference-in-differences.

We describe two of the methods that have been proposed to deal with the challenge of heterogeneous treatment effects and staggered treatment adoption. The first, proposed by Callaway & Sant’Anna,^8^ focuses on dealing with heterogeneity and staggered adoption of treatment in real world settings by estimating separate cohort-period effects. The second is the extended two-way fixed effects estimator proposed by Wooldridge,^9^ which allows for heterogeneity by including additional interaction terms in the single, aggregate TWFE regression model.

We then give briefer guides to some other proposed estimators, considering which problems they solve, how they solve them and what they require. We conclude with a set of considerations for deciding which estimation procedure to use in which situations.

Our aim is to present the issues in a non-technical manner. We provide references for further reading and for practical materials on how to code the various estimation methods at the end.

## Results

### Challenges for the simple difference-in-differences method

#### Fundamental features of the simple 2×2 design

Box 1 provides the classic description of the difference-in-differences method. It is based on two groups (one that becomes exposed and the other that remains unexposed) and two periods (before and after the exposed group becomes exposed). In this situation, providing the fundamental assumptions including the ‘parallel trends’ assumption are valid, and the difference between the change over time in the exposed group and the change over time in the unexposed group is a valid estimate of the treatment effect. Returning to our previous example, this could mean evaluating the short-term mental health effects of the introduction of Universal Credit using data on an exposed and an unexposed geographical area for 2013 and 2014 only.

#### Deviations from the 2x2 design

The evaluation of policies in real world settings typically deviate from the simple 2×2 setting. This complexity has led researchers to calculate difference-in-differences in a variety of ways (see, eg, the variety of methods used to evaluate Universal Credit in table 1). This variety prompts the question: what led authors to use such different approaches? What real-world challenges to difference-in-differences were they trying to overcome?

**Table 1:** Approaches taken to evaluating mental health effects of the Universal Credit welfare reform

Study	Definition of exposed (E) and unexposed (U) groups	Timing	Estimators used
Wickham *et al*^2^	**T:** Unemployed adults **C:** Employed adults	Staggered	*Main analysis*: Standard DiD with multiple time periods
Thornton and Iacoella^30^	**T:** T1=Individuals receiving UC T2=individuals receiving a legacy benefit T3=Individuals receiving a different benefit **C:** Individuals receiving no benefits	Two periods	*Main analysis:* TWFE *Additional analysis:* Standard 2×2 DiD
Song *et al*^31^	**T:** Children with an unemployed parent **C:** Children with no unemployed parents	Two periods	*Main analysis:* Standard DiD with multiple periods (but homogenous treatment timing). *Additional analyses*: Standard 2×2 DiD
Brewer *et al*^32^	Employed individuals (specific criteria) **E:** In areas where UC was available **U:** In areas where UC not yet available	Staggered rollout	*Main analysis:* TWFE *Additional analysis:* Followed principles of Callaway and Sant’Anna (2021)
Marimpi *et al*^33^	**E:** Individuals on UC or any legacy benefit **U:** Individuals on any other State benefit	Staggered rollout	*Main analysis:* TWFE and Local Projections DiD (Dube et al., 2025)
Baxter *et al*^34^	Low-income individuals **E:** In areas where UC was available **U:** In areas where UC not yet available	Staggered rollout	*Main analysis*: Two-stage DiD (Butts and Gardner, 2021; Gardner, 2022) *Additional analyses:* TWFE to test for bias
Amo-Agyei *et al*^35^	**E:** Areas in which UC was available **U:** Areas in which UC not yet available	Staggered rollout	*Main analysis:* Callaway & Sant’Anna (2021) *Additional analyses*: Range of other estimators. TWFE included to illustrate bias.
Sutton *et al*^36^	Product of area-level take-up of UC and individual-level probability of claiming welfare benefits	Continuous time (2015–2019)	*Main analysis:* New interactions method proposed in the paper

DiD, difference-in-differences; TWFE, two-way fixed effects; UC, Universal Credit.

#### What problems do these deviations cause?

The principal deviations from the classic 2×2 situation are listed in Box 2.

Box 2 **Deviations from the classic difference-in-differences situation**

- Inclusion of measured confounders that vary over time
- Multiple periods after the introduction of the treatment
- Differential timing of when units become exposed
- Treatment effects that vary over time
- Treatment effects that vary across exposed units

A common way to estimate a single overall effect of an exposure is to use a two-way fixed effects (TWFE) model that includes indicators for the exposed units and the post-exposure period.^2 9^ Alternatively, researchers may use event study designs to estimate how treatment effects evolve after the exposure.^10 11^ This latter approach produces separate estimates for each post-exposure period.

There are three fundamental problems that may arise with these approaches to difference-in-differences:

##### Problem 1: Requirement for parallel trends in all post-exposure periods

For the difference-in-differences estimates to be valid, the researcher has to assume that parallel trends must hold across all post-exposure periods. This is harder to justify over longer follow-up periods, and it makes these estimates less reliable than those in shorter studies, where there is less risk of unobserved factors that differ by group having time-varying impacts on outcomes. This issue affects both event studies and TWFE models.

##### Problem 2: Bias from non-identical trends across multiple pre-exposure periods

Even if the parallel trends assumption holds in the post-exposure period and the exposure starts at the same time for all exposed units, estimating one overall effect using TWFE with multiple pre-intervention periods can be misleading when the trends prior to the start of the exposure are not identical. For example, if exposed units show some upward divergence from the controls before the intervention and then experience small negative effects from the exposure, the TWFE estimate can exaggerate the true effect. Even if the divergence in trends in the period before exposure is not statistically significant, changes in post-exposure outcomes due to pre-existing trends may be wrongly attributed to effects of exposure.

##### Problem 3: Staggered treatment timing and heterogeneity

Exposures often begin at different times for different groups. If the treatment effects are the same in all contexts, this is not a problem. But when effects vary by group, calendar time or exposure duration, aggregation becomes critical. Each cohort (defined by timing of exposure) is effectively a separate treatment group. Ignoring this and using standard TWFE or event study models has been shown to distort the results.^8^ Evaluation methods that do not explicitly take account of the staggered nature of the introduction of some exposures have been shown to use already-treated units implicitly as controls for later-treated units. In such settings, Goodman-Bacon^12^ demonstrated that the overall difference-in-difference TWFE estimate can be decomposed as a weighted average of all possible 2×2 difference-in-differences comparisons. The weights that each comparison receives are determined by group size and variation in exposure status. In some situations, these weights can be negative, sometimes resulting in different signs for the combined effect compared with the signs of the effects for some or, in extreme cases, all cohorts.

Event study models can also suffer bias when treatment effects vary across cohorts, misrepresenting how effects change over time.^10^ The upshot is that staggered introduction of an exposure requires careful treatment-control comparisons and handling of cohort-specific effects because ignoring heterogeneity in the effect of the exposure across cohorts can lead to severe distortions.

### Two new approaches to difference-in-differences estimation

Here, we outline two methods that have sought to address some of these challenges in different ways, one that is based on disaggregated analysis and doubly robust estimation, and another that introduces more flexibility into a single aggregate TWFE regression model. In a later sub-section, we describe other approaches that have been proposed.

#### The Callaway and Sant’Anna approach

One widely used solution to avoid the perils of standard approaches to estimate difference-in-differences with staggered treatment timing and heterogeneous effects is the approach proposed by Callaway and Sant’Anna (hereafter, the CS method).^8^ This involves four steps:

##### Step 1. Defining the right comparisons

CS emphasises the importance of choosing appropriate comparison groups. The simplest approach is to compare treated units to those that are never treated. If that is not possible, or if the never-treated units differ substantially from the treated ones, units that have not yet been treated can be used as comparators.

##### Step 2. Breaking the problem into building blocks

Rather than estimating one overall effect, CS breaks the evaluation into smaller pieces. It looks at treatment effects for each cohort (units treated at the same time) in each time period as in the simple 2×2 situation. For example, if some units are treated in year 2 and others in year 3, CS estimates the effect for each group at each point after treatment separately. This creates a set of “building blocks” that reflect how effects evolve over time and across cohorts.

##### Step 3. Doubly robust estimation

To estimate the effects, Callaway and Sant’Anna^8^ favour a ‘doubly robust’ estimator. This approach combines two models, one for exposure and one for outcomes, and the estimates are valid as long as at least one of those models is correct. Compared with a single regression model for the outcome, this reduces the risk of bias from incorrect model specification (eg, specifying a linear model for a non-linear relationship). It also allows straightforward adjustment for covariates when the parallel trends assumptions hold only when conditioning on these covariates, though this requires good overlap in the distributions of the covariates between the exposed and unexposed units.

##### Step 4. Aggregating results intentionally

As CS produces an estimate for each combination of cohort and time period, researchers need to decide how to combine them to produce a summary of the results. They may, for example, want to produce a single summary effect or they may be more interested in summarising how the effects change over time. Callaway and Sant’Anna^8^ encourage defining a weighting scheme that matches the research question, for example, averaging across all cohorts or focusing on a specific time since the introduction of treatment. Aggregations that are commonly required can be obtained relatively straightforwardly in software such as Stata (using *xthdidregress aipw*) and R (using *did*).

### Extended two-way fixed effects (ETWFE)

In contrast to CS, Wooldridge^9^ suggested an extended specification of the two-way fixed effects model (called ETWFE) that retains the simplicity of single-equation regression-based approaches while addressing concerns about heterogeneity in treatment effects across treatment cohorts, groups and exposure time.

The baseline TWFE model is extended by adding a full set of cohort-time interactions so that each treated group is compared with an appropriate control group at each time point. Average values of variables can be included for each unit over time and for each time period over units to correct for correlation between these variables and the two sets of fixed effects. Importantly, within-unit and within-time averages of the treatment indicator can be included to allow for staggered adoption.

The full set of cohort-time interactions separates out the variation due to forbidden comparisons (eg, newly treated units being compared with already-treated units) that cause the bias in the TWFE model without appropriate interactions. This approach provides valid treatment effects under the standard assumptions of parallel trends and no anticipation, meaning units do not change behaviour before treatment occurs.^9^ By including interactions and average values of the treatment indicator over units and over time, the estimator allows for heterogeneity in treatment effects and timing while maintaining computational simplicity.

The primary strength of this framework is its transparency and familiarity to researchers who have used the TWFE approach. Researchers can implement ETWFE using standard regression software (*xthdidregress twfe* in Stata and *etwfe* in R), making it attractive for applied work in public health and related fields. The method naturally extends to analyses of effects that vary over time, such as event studies with leads and lags, enabling checks on whether there are differential trends in the outcomes prior to introduction of the intervention and investigation of the trajectories of the treatment effects after introduction. The approach can also be seen as nesting a wide range of different estimators as special cases in a single, fully flexible regression model, reducing confusion about competing methods and clarifying the conditions under which TWFE remains valid.^9^

Perhaps the primary limitation of ETWFE is that it requires careful specification because omission of necessary interactions can lead to biased estimates. In addition, while it is relatively straightforward computationally, the method is parametric and assumes a correctly specified linear model, which may be restrictive in settings with nonlinear outcomes or complex functional forms. The method is also less transparent about how much weight is given to particular units or time periods when aggregate treatment effects are generated.

### Other considerations for difference-in-differences estimation

The CS and ETWFE approaches are just two of the new approaches to difference-in-differences estimation that have been proposed. In this sub-section, we discuss some additional considerations and refer to other approaches that may be needed to address these. Table 2 provides summary details for some of these alternative approaches.

**Table 2:** List of selected new estimators

Estimator	Authors	Year	Key contribution vs standard DiD
Callaway & Sant’Anna Estimator	Callaway, Sant’Anna^3^	2021	Group-time ATT estimates for staggered adoption; flexible aggregation and heterogeneity handling.
Sun & Abraham Event-Study	Sun, Abraham^5^	2020	Corrects bias in TWFE event-study designs; uses interaction-weighted approach for heterogeneity.
did2s (two-stage DiD)	Butts, Gardner^37^	2022	Two-stage imputation estimator; robust under staggered timing and heterogeneity.
Imputation Estimator	Borusyak, Jaravel, Spiess^11^	2021	Avoids negative weighting in TWFE; imputes untreated potential outcomes for staggered adoption.
Reweighting Estimator	de Chaisemartin, D’Haultfoeuille^38^	2020	Reweights comparisons to eliminate bias from heterogeneous treatment effects and timing variation.
Simple Approaches to Nonlinear DiD	Wooldridge^29^	2023	Extends DiD to nonlinear models (logit, Poisson) using pooled quasi-MLE; handles binary, fractional, and count outcomes under staggered interventions.
Local Projections	Dube *et al*^39^	2025	Adapts a time-series local projections technique to produce dynamic heterogeneous treatment effects.
Two-Way Fixed Effects, Two-Way Mundlak Regression	Wooldridge^4^	2025	Shows equivalence between TWFE and two-way Mundlak regression; provides flexible pooled OLS framework for heterogeneous treatment effects and event-study estimation.

ATT, average treatment effect on the treated; MLE, maximum likelihood estimation; TWFE, two-way fixed effects.

#### When the assumption of parallel trends is implausible

With multiple pre-intervention periods, if the assumption that exposed and unexposed groups would have followed similar trends without the exposure seems unrealistic, one option is to adjust for covariates so that the parallel trends assumption holds after conditioning on these variables. This often involves adding interactions between time and group indicators.^13^ However, this assumes that unobserved factors influence the outcomes linearly in both groups over time, which is a strong assumption.

More flexible approaches, such as interactive fixed effects models,^14^ use latent factors to capture unobserved heterogeneity. These can help but may produce biased estimates when treatment effects vary across units.^15^ There are other more advanced methods that can deal with non-parallel trends, including augmented synthetic controls, generalised synthetic controls and synthetic DiD.^16–18^

#### Continuous treatments

Standard difference-in-differences assumes a binary treatment, but many interventions vary in intensity in practice. The usual two-way fixed effects approach does not yield a clear causal interpretation when continuous measures of dose are interacted with time. Alternative estimators have been proposed for these cases.^19 20^

#### Looking at the full distribution of treatment effects

The treatment effect estimated by difference-in-differences is typically the average treatment effect on the treated. In some situations, the researcher is more interested in the distribution of treatment effects. The change-in-changes method^21^ addresses this by mapping the pre-exposure outcomes of exposed units to equivalent positions in the control group’s distribution and then projecting forward to obtain a counterfactual distribution. This allows researchers to estimate how the treatment effects vary across the outcome distribution.

#### When control groups are unavailable

Sometimes all units are exposed, leaving no unexposed group for comparison. In this case, standard difference-in-differences cannot estimate the counterfactual. In staggered designs, researchers can use ‘not-yet-treated’ units as controls. This is possible for all cohorts except the cohort who are last to be exposed. Another option is to exploit variation in exposure intensity across groups, known as a fuzzy design.^22^ A further solution is to use a ‘negative outcome control’, an outcome unaffected by the intervention but subject to the same confounding as the main outcome.^23^ If such an outcome is not available, researchers can reframe the evaluation question to compare effects across subgroups defined by other characteristics.^24^

#### Outcome type matters

The standard difference-in-differences approach involves comparisons of mean values, which can be problematic for outcomes where linear models are arguably inappropriate, such as limited (eg, binary) or skewed (eg, count) outcomes. Researchers must consider the scale on which the parallel trends assumption holds, whether on the observed scale or a transformed one.^25^ For example, if trends are parallel once the outcome is log transformed, they are not parallel on the original unlogged scale. Additive effects on one scale may imply multiplicative effects on another.^26^ Wooldridge^27^ proposes estimators for outcomes in the linear exponential family (normal, binomial, Poisson), assuming parallel trends on the transformed scale. These methods also work when there is staggered adoption of an intervention.

## Conclusion

Difference-in-differences has become a popular method in applied population health studies. The fundamental principles of difference-in-differences are easy to understand in the classic situation of two groups and two time-periods. In this paper, we have highlighted some recent literature on problems and solutions when the setting is more complex than the classic 2×2 design. Many of these complexities (multiple exposed groups, multiple time periods and/or exposure at different times) are very common in population health research, such as when researchers have sought to identify the mental health effects of a national welfare reform. Box 3 provides a guide to what researchers should consider when using a difference-in-differences approach to evaluation.

Box 3 **Choosing a difference-in-differences method**

1. Decide what to estimate. A key choice is between a single, summary effect of exposure or effects of exposure that may be different across cohorts and/or time-periods.
2. Staggered adoption and heterogeneity. If the time that exposure begins varies across units, and effects of exposure differ across cohorts and/or by the duration of exposure, simple TWFE and event-study methods are likely to be biased.
3. Aggregation and reporting. When aggregating across different cohort–time effects, choose weights that match the policy question.
4. Parallel trends and anticipation. Scrutinise the parallel trends assumption and be explicit whether it is assumed only once conditioned on other variables. Provide supporting evidence based on trends during pre-exposure periods and look for evidence of anticipation. If the parallel trends assumption is not supported, consider alternative methods such as interactive fixed effects and synthetic control methods.
5. Comparison groups and spillovers. Avoid ‘forbidden’ comparisons to already-treated units. Be explicit over choice of never-treated or not-yet-treated units as controls. Consider potential for spillovers, redefining exposure or using estimators that accommodate spillovers if necessary.
6. Outcome measurement scale. For binary, count or skewed outcomes, ensure that the parallel trends assumption applies and results are reported on the appropriate scale. A useful resource is Wooldridge’s^29^ family of exponential approaches for nonlinear DiD with staggered interventions.
7. Continuous treatments (dose-response). If exposure intensity varies, use DiD estimators for continuous treatment^20 21^ and verify that there is sufficient overlap.
8. No untreated group available. Use not-yet-treated units, fuzzy DiD, or negative outcome controls. Alternatively, reframe the question to focus on subgroup contrasts.
9. Inference and clustering. Use standard errors that are robust to clustering at the level of assignment to the exposure, especially if there are few clusters.
10. Software and implementation. Both CS and ETWFE can be implemented in standard software such as R or Stata. Practical code resources are available for most alternatives to standard difference-in-differences, and they show sensitivity of the results to a range of difference-in-differences methods.

There are several recent papers that have reviewed and compared different approaches to difference-in-differences in the econometric literature^4 5^ and in health journals.^6 7^ We have focused primarily on two methods, taking different approaches, to explain intuitively what these new methods are seeking to do and have signposted to other approaches that have been proposed.

There are several other recent developments that are important to consider when using difference-in-differences approaches that are beyond the scope of this article. These include newer ways to consider whether the fundamental assumption of parallel trends in the exposed and unexposed groups is plausible^28^ and how to adjust standard errors for clustering of observations by their exposure.^29^

The recent interest in the limitations of difference-in-differences and the development of methods to take account of real-world complexities highlight the need for population health researchers to test the fundamental assumptions of the methods they use and demonstrate the robustness of the results to different methodological approaches.

## Data Availability

Data sharing not applicable as no datasets generated and/or analysed for this study.
